# Intraoperative use of dexmedetomidine promotes postoperative sleep and recovery following radical mastectomy under general anesthesia

**DOI:** 10.18632/oncotarget.18157

**Published:** 2017-05-24

**Authors:** Cunxian Shi, Jin Jin, Qiang Pan, Shan Song, Kezhong Li, Jiahai Ma, Tao Li, Zhi Li

**Affiliations:** ^1^ Department of Anesthesiology, The Affiliated Yantai Yuhuangding Hospital of Qingdao University, Yantai, Shandong, P.R. China; ^2^ Department of General Surgeon, Rushan People’s Hospital, Rushan, Yantai, Shandong, P.R. China

**Keywords:** dexmedetomidine, radical mastectomy, sleep disturbance, recovery, fatigue

## Abstract

Postoperative sleep disturbance and fatigue following radical mastectomy were high risks for prolonged convalescence in patients with breast cancer. The present study was designed to observe the effect of intraoperative use of dexmedetomidine on postoperative sleep, fatigue and recovery following radical mastectomy under general anesthesia. Forty-seven patients were randomized into two groups that were maintained with propofol/remifentanil/Ringer’s solution (Control group), or propofol/remifentanil/Dexmedetomidine (DEX group) for surgery under general anesthesia. During the first night following surgery, patients receiving dexmedetomine spent more time sleeping when compared with those form the Control group. During the first week following operation, when compared with the Control group, patients from the DEX group had a higher score of global 40-item recovery questionnaire on day 3 following operation, and lower 9-question fatigue severity scores on day 3 and day 7 following operation. In conclusion, intraoperative use of dexmedetomidine is sufficient to improve postoperative sleep disorder, promote postoperative recovery. The adverse effect of dexmedetomidine on sleep disturbance might be contributed to its recovery-promoting effect.

## INTRODUCTION

Postoperative sleep disturbance and fatigue are high risk factors to prolonged convalescence and additional hospital costs. Recent studies reported that aesthesia management may affect postoperative outcomes to promote recovery. For example, clinical trials have found that intraoperative use of dexmedetomidine for general anesthesia, a highly selective alpha-2 adrenergic agonist, was able to attenuate postoperative fatigue, promote recovery, facilitate the analgesic property of PCA morphine, reduce morphine consumption as well as its related adverse effects [1-11]. A more recent study indicated that low-dose infusion of dexmedetomidine are able to promote sleep quality in nonmechanically ventilated elderly patients in the ICU following operations [12]. Dexmedetomidine was also reported to improve postoperative outcomes in breast cancer patients following operations, such as displaying analgesia-promoting effect on acute and chronic pain [4, 10, 11].

Based on the evidence above, we hypothesized that intraoperative use of dexmedetomidine might promote the sleep quality and recovery in breast cancer patients following radical mastectomy under general anesthesia.

## MATERIALS AND METHODS

### Participants

This study was approved by the Institutional Medical Ethics Committee of The Affiliated Yuntai Yuhuangding Hospital of Qingdao University, and was in accordance with the approved guidelines. Written informed consent was obtained from each patient. The sample size of the study was calculated according to previous studies, and was based on a pilot study. Twenty patients in each group were required to detect a difference of sleep time (primary outcome) with a power of 0.8 and type I error of 0.05. To compensate for dropouts and deviation from normality, a total of 56 patients scheduled for radical mastectomy under general anesthesia were assessed for eligibility. We targeted an 80% probability (β = 0.2) with a significance level of 0.05 and a ∼10% dropout rate. Finally, forty-seven patients enrolled were analyzed in Control (*n* = 23), and DEX (*n* = 24) based on their treatment. Patients received propofol, remifentanil, and Ringer’s solution or dexmedetomidine for general anesthesia maintenance, respectively. To be blind, the maintenance syringe pump was prepared by a different anesthesiologist. NRS score evaluated by another different anesthesiologist(see supplementary file-2). Total sleeping time was recorded following operation starting form 8:00PM to 8:00 AM. Patients matching the following criteria were included in this study: American Society of Anesthesiologists (ASA) grade II or III; between 30 and 60 years old; weight 45-75 kg; height 145-175 cm. Patients were excluded if they had a history of opioid addiction, long-term alcohol abuse or smoking history, current use of sedative-hypnotic drug(s); obesity (BMI > 30); sleep disorders, postoperative nausea and vomiting history; or neuropsychiatric diseases and related treatment history.

### Anesthesia

Before induction, patients from the DEX group received a fast infusion of 100 ml Ringer solution with or without DEX (1μg/kg) as a loading dose within 15 min. For induction, patients from both groups received midazolam (0.05 mg/kg), remifentanil (2-5 μg/kg), propofol (1.5-2 mg/kg), and cisatracurium (0.2 mg/kg). Immediately after intubation, the patients were ventilated with an oxygen and air mixture (FiO2 = 0.4) with a PetCO2 of 30-35 mmHg. Intravenous infusion was switched to a maintenance syringe pump at rate of 50-80 μg/kg/min for propofol, 0.15-0.2 μg/kg/min for remifentanil, and 0.4 μg/kg/h for dexmedetomidine. Cisatracurium (0.05 mg/kg) was intermittently used for muscle relaxation. Patients were transferred to the PACU after extubation.

### Statistics

All data were presented as mean ± SD. or the exact values, and analyzed with GraphPad Prism 6.0 software. Age, weight, height, blood pressure, heart rates, operation time, anesthesia time, PACU time and sleep time were compared with unpaired *student’s t test*. Recovery and fatigue scores at different time points were compared between the two group with two-way *ANOVA* followed by *Bonferroni post-test*. ASA grade and postoperative adverse effects were analyzed with *Fisher’s test. P* < 0.05 were considered to be significant.

## RESULTS

### Demographic data of participants

**Figure 1:**
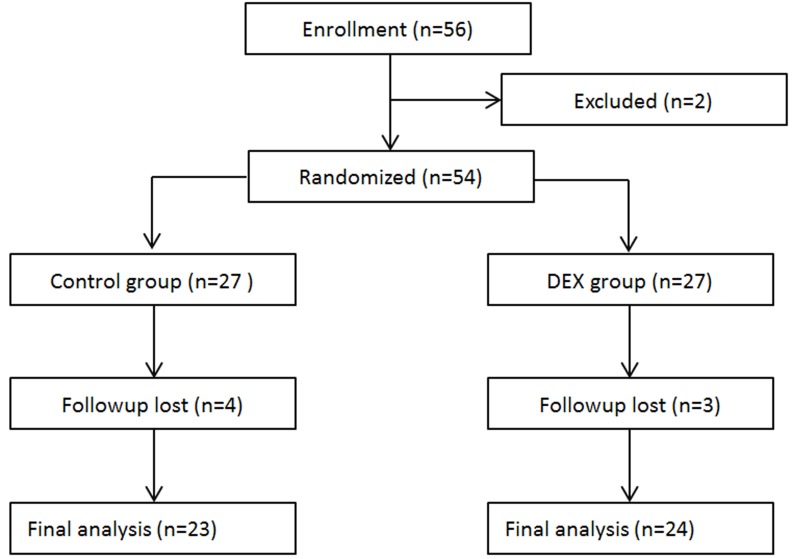
Flow diagram of the study

A total of 56 patients scheduled for radical mastectomy under general anesthesia were assessed for eligibility. Nine patients were excluded due to ineligibility. Finally, 47 patients were enrolled in this clinical observation trail and randomized into two groups: Control group (23 patients) and D group (24 patients), which received either propofol/remifentanil and Ringer’s solution or dexmedetomidine (Figure 1). Patients from the two groups were comparable with respect to age, weight, height, BMI, ASA class, baseline MBP, 24 hour MBP, baseline HR, 24 hour heart rate, operation time, anesthesia time, and PACU time (Table 1).

**Table 1:** Basic demographic data and surgery duration (mean ± SD).

Variables	Control (*n* = 23)	DEX (*n* = 24)	Values of *P*
Age (years)	47.74 (8.735)	49.17 (8.458)	0.572
Weight (Kg)	53.46 (14.85)	51.49 (11.18)	0.609
Height	161.10 (6.324)	161.3 (6.587)	0.930
BMI (Kg/m2)	26.44 (2.546)	25.58 (2.325)	0.235
ASA II/III	17/6	21/3	0.287
Baseline MBP (mmHg)	83.41 (4.758)	83.52 (5.399)	0.944
24 hour MBP (mmHg)	89.36 (7.832)	88.67 (8.977)	0.7812
Baseline HR (beats/min)	70.57 (7.948)	73.25 (6.469)	0.2091
24 hour HR (beats/min)	70.57 (7.948)	73.25 (6.449)	0.5081
Operation time (min)	140.30 (18.67)	134.0 (16.05)	0.214
Anesthesia time (min)	168.10 (22.78)	166.9 (22.51)	0.855
PACU time (min)	43.70 (4.733)	42.04 (4.912)	0.0447

### Postoperative sleep time of the two groups

**Figure 2:**
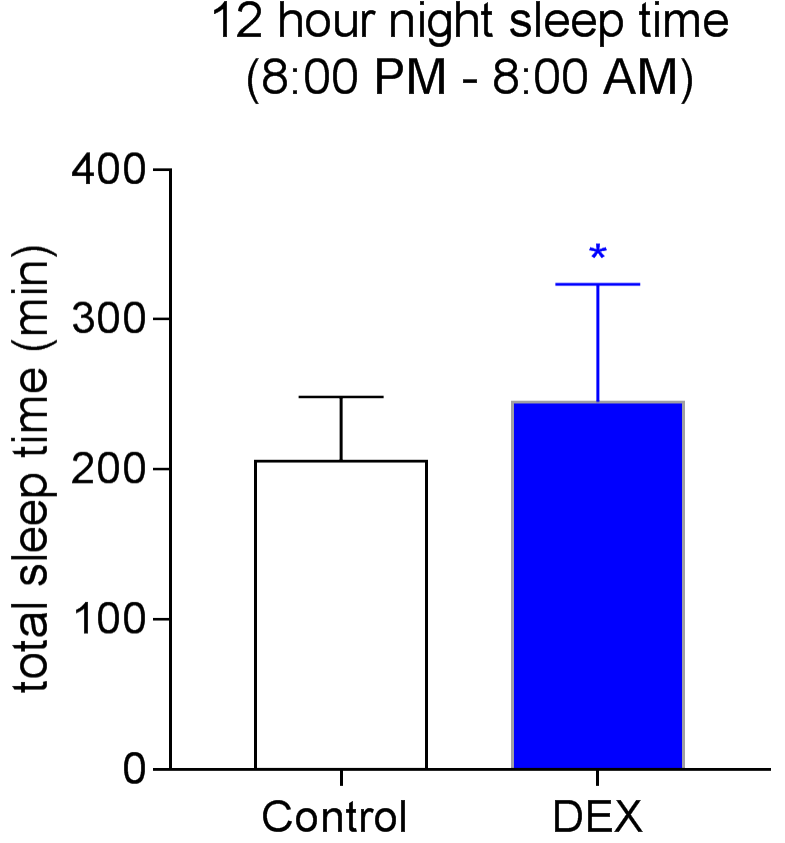
Postoperative 12 hour night sleep time from 8:00 PM to 8:00 AM *P = 0.0381.

Postoperative sleep time was monitored from 8:00 PM on the operation day to next 8:00 AM for 12 hours. During first 12 hours, patients from the DEX group had a longer sleep time as compared with patients from the Control group (Figure 2).

### Postoperative recovery and fatigue evaluation

**Figure 3:**
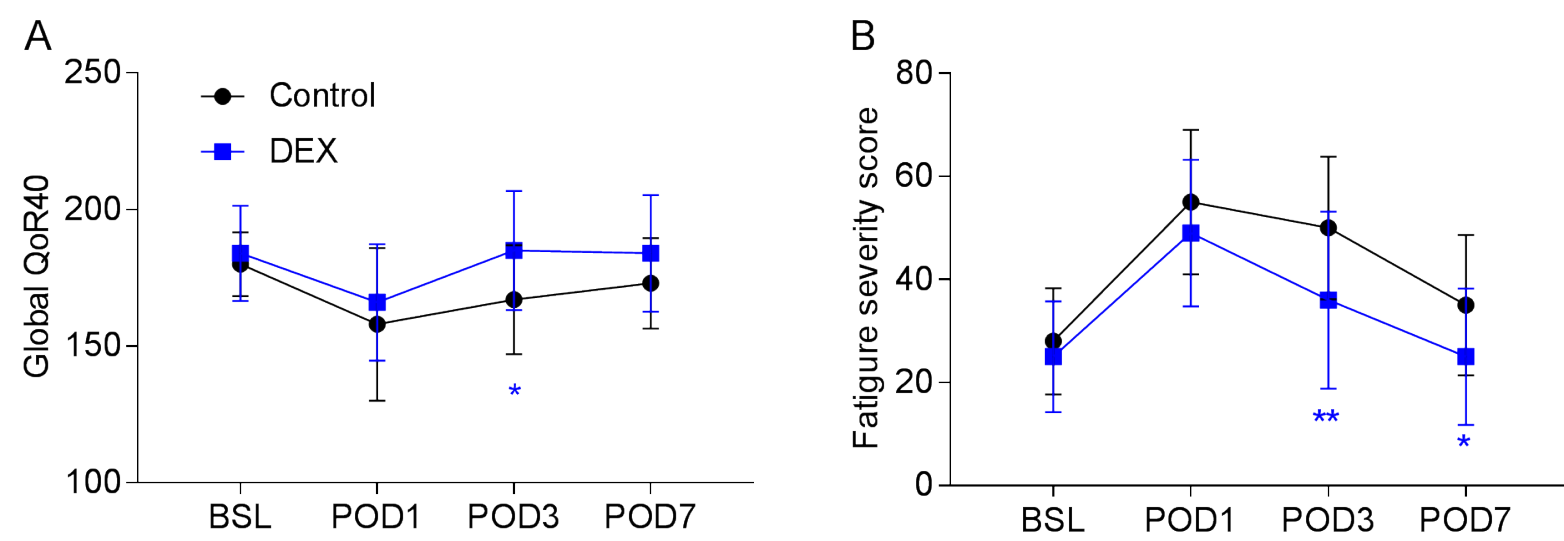
Postoperative recovery in the two groups **A.** Global 40-item quality of recovery questionnaire evaluation, *P < 0.05. **B.** Nine question fatigue scores, *P < 0.05, **P < 0.01. BSL: baseline, POD: post-operative day.

Postoperative recovery was evaluated with the global 40-item quality of recovery questionnaire. Lower scores were observed on day 1 following operation in the two groups. At POD 3 time point, patients in the DEX group shown significantly higher scores compared with their Control group. Patients from the two groups both recovered to their baseline level at POD 7 time point (Figure 3A).

Similarly, Patients in the DEX group had a lower fatigue severity score than those in the Control group at both POD3 and POD7 time points (Figure 3B).

### Postoperative adverse effects

A decreased incidence vomiting and a trending decrease of nausea were observed during the first 24 hours in the patients from DEX group when compared with those from the Control group (Table 2). No difference was observed in other adverse effects between the two groups.

**Table 2:** Adverse effects.

Variables (+/-)	Control (*n* = 23)	DEX (*n* = 24)	Values of *P*
Nausea	13/10	8/16	0.1468
Vomitting	11/12	6/18	0.0392
Bradycardia	3/20	4/20	>0.9999
Respiratory depression	0/23	0/24	>0.9999
Itching	2/21	1/23	0.6085

+/-: positive patient number/ negative patient number.

## DISCUSSION

The present study have found that general anesthesia maintained with dexmedetomidine improved sleep disturbance and promote recovery following radical mastectomy in patients with breast cancer. The adverse effect of dexmedetomidine on sleep disturbance might be contributed to its recovery-promoting effect.

Radical mastectomy under general anesthesia is a widely-used therapy in patients with breast cancer [13-15]. It has been known that breast cancer patients undergoing radical mastectomy experience severe sleep disturbance and fatigue [16, 17], which might lead to prolonged convalescence and additional hospital costs. There has been a pursuit for novel drugs or for more information regarding combining the currently-available drugs to alleviate these symptoms. Dexmedetomidine is a alpha-2 receptor agonist developed in the 1990s, and it was first introduced into hospital as a sedative in ventilated patient in the intensive care unit [18]. Unexpectedly, it emerged as an adjuvant of local/general anesthetics and opioids for local and general analgesia [19-21] to improve analgesic effects of local anesthetics or general anesthesia.

Clinical trails have recently reported that intraoperative use of dexmedetomidine promoted morphine’s analgesic property in patient-controlled analgesia and postoperative recovery, which might contribute to the recovery-promoting effect of dexmedetomidine following different kinds of operations [1, 6, 22]. In the present study, we further found a novel effect of intraoperative use of dexmedetomidine for general anesthesia maintenance on sleep disturbance: during the first 12 hours of night sleeping, patients from the DEX group spend more time sleeping when compared with the Control groups, which might be a contributor to the recovery-promoting effect of dexmedetomidine. Consistent with recent studies from different centers [6, 23-25], using a global 40-item questionnaire, we observed that the Global QoR-40 score was significantly improved in the DEX group on day 3 after surgery. All of the patients shown higher fatigue level after surgery, but on day 3 and day 7 after surgery, patients in the DEX group reported significantly lower scores for fatigue severity than their controls. Many factors are responsible for slow recovery from surgery, including postoperative acute pain, fatigue, and surgery-induced metabolic, endocrine, and immune changes known as ‘stress responses’. Recently, Dong-Jian Ge and colleagues proposed that the inhibitory effect of dexmedetomidine on the vicious cycle among surgery-induced stress responses, fatigue and acute pain to interpret the recovery-promoting effect of dexmedetomidine [6]. Poor sleep quality was associated high fatigue severity and postoperative stress response [26, 27]. Studies from another group in China recently reported that low-dose dexmedetomidine infusion may improve overall sleep quality in nonmechanically ventilated elderly patients in patients following noncardiac surgery [28, 29].

Thus, improved sleep quality/time might be helpful to reduce fatigue and postoperative stress responses. Collectively, we believe that together with its reversive effect on sleep disturbance, the proposal above will better interpret the recovery-promoting effect of intraoperative use of dexmedetomidine.

There might be limitations in the present study: 1) The study was performed only in female patients, 2) For the observation of sleep conditions, we only studied the total sleep time and did not look at different subtypes of sleep, such as rapid and non-rapid eye movement sleep, 3) To exclude the effect of diurnal rhythm, the total recording time of sleep started from 8:00P M to 8:00 AM, however, the operation time was variable among patients.

Collectively, this study found that general anesthesia combined with dexmedetomidine was useful for promoting postoperative recovery in breast cancer patients following radical mastectomy. Furthermore, the adverse effect of dexmedetomidine on sleep disturbance might be a novel contributor to interpret the recovery-promoting effect of intraoperative use of dexmedetomidine.

## SUPPLEMENTARY MATERIALS AND TABLE




