# Supplementary material for: Intraoperative use of dexmedetomidine promotes postoperative sleep and recovery following radical mastectomy under general anesthesia
Source: Oncotarget. 2017 May 24;8(45):79397–403. doi: 10.18632/oncotarget.18157 (PMC5668051; doi:10.18632/oncotarget.18157)
Supplement: Supplementary file 1 [file oncotarget-08-79397-s001.pdf]

Intraoperative use of dexmedetomidine promotes postoperative sleep and recovery following radical mastectomy under general anesthesia

Supplementary Material

For Supplementary Table see in Supplementary Information

The Numeric Pain Rating Scale Instructions

General Information:

- The patient is asked to make three pain ratings, corresponding to current, best and worst pain experienced over the past 24 hours.
- The average of the 3 ratings was used to represent the patient's level of pain over the previous 24 hours.

Patient Instructions (adopted from (McCaffery, Beebe et al. 1989):

"Please indicate the intensity of current, best, and worst pain levels over the past 24 hours on a scale of 0 (no pain) to 10 (worst pain imaginable)"

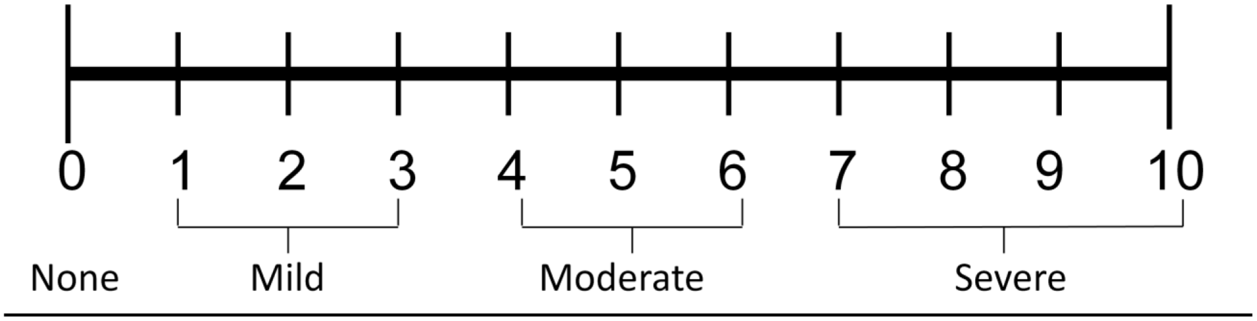

REFERENCE

McCaffery M, Beebe A, Donovan MI. (1989). Pain: Clinical manual for nursing practice, Mosby St. Louis, MO.
